# Lignocellulose-Based Superabsorbent Polymer Gel Crosslinked with Magnesium Aluminum Silicate for Highly Removal of Zn (II) from Aqueous Solution

**DOI:** 10.3390/polym13234161

**Published:** 2021-11-28

**Authors:** Yuhong An, Wanqi Zhang, Hui Liu, Yuan Zhong, Zichu Hu, Yali Shao, Zhangjing Chen, Yukun Ren, Boyun Wang, Sunguo Wang, Xiaotao Zhang, Ximing Wang

**Affiliations:** 1 College of Material Science and Art Design, Inner Mongolia Agricultural University, Hohhot 010018, China; anyuhong@emails.imau.edu.cn (Y.A.); nmgndcyyzwq@emails.imau.edu.cn (W.Z.); nndcyylh@emails.imau.edu.cn (H.L.); zhongyuan@emails.imau.edu.cn (Y.Z.); shaoyali_lucky@emails.imau.edu.cn (Y.S.); 2 College of Science, Inner Mongolia Agricultural University, Hohhot 010018, China; huzichu@emails.imau.edu.cn (Z.H.); 13485473614@emails.imau.edu.cn (B.W.); 3 Department of Sustainable Biomaterials, Virginia Polytechnic Institute and State University, Blacksburg, VA 24060, USA; chengo@vt.edu; 4 Bioimaging Research, Sanofi Global R&D, Framingham, MA 01702, USA; yukun.ren@sanofi.com; 5 Sungro Bioresource & Bioenergy Technologies Corp., Edmonton, AL T6R3J6, Canada; wangsunguo@gmail.com; 6 Inner Mongolia Key Laboratory of Sandy Shrubs Fibrosis and Energy Development and Utilization, Hohhot 010018, China

**Keywords:** nano-lignocellulose, magnesium aluminum silicate, intercalation, adsorption, Zn (II)

## Abstract

Lignocellulose (LCE) was ultrasonically treated and intercalated into magnesium aluminum silicate (MOT) clay to prepare a nano-lignocellulose magnesium aluminum silicate polymer gel (nano-LCE-MOT) for the removal of Zn (II) from aqueous solution. The product was characterised using nitrogen adsorption/desorption isotherm measurements, Fourier-transform infrared spectroscopy, scanning electron microscopy and energy-dispersive X-ray spectroscopy. The conditions for the adsorption of Zn (II) on nano-LCE-MOT were screened, and adsorption kinetics and isotherm model analysis were carried out to explore the adsorption mechanism and achieve the optimal adsorption of Zn (II). Optimal adsorption was achieved at an initial Zn (II) concentration of 800 mg/L at 60 °C in 160 min at a pH of 4.52. The adsorption kinetics were explored using a pseudo-second-order model, with the isotherm adsorption equilibrium found to conform to the Langmuir model. The maximum adsorption capacity of the nano-LCE-MOT polymer gel toward Zn (II) is 513.48 mg/g. The materials with adsorbed Zn (II) were desorbed using different media, with HCl found to be the most ideal medium to desorb Zn (II). The optimal desorption of Zn (II) was achieved in 0.08 mol/L HCl solution at 65 °C in 60 min. Under these conditions, Zn (II) was almost completely desorbed from the adsorbents, with the adsorption effect after cycling being slightly different from that of the initial adsorption.

## 1. Introduction

Heavy metals that are released into the environment tend to persist indefinitely, circulate and eventually accumulate throughout the food chain, posing a serious threat to the environment, animals and humans [1,2,3]. Even at relatively low concentrations, heavy metals are toxic to aquatic flora and fauna, and in some of these organisms they are assimilated, stored and concentrated. In the presence of zinc sulphides, carbonates, silicates, and oxides are formed in Nature. The presence of Zn (II) at pH < 7 is negligible, but at pH > 7 it is present in different forms. Zn (II) is discharged in the wastewater of the waste streams of metal, chemical and pulp and paper processes, from galvanised steel plant lines, plant zinc and brass metal processes, Zn (II) and brass plating and processes such as viscose rayon fibre production. The World Health Organization recommends a maximum acceptable zinc concentration limit in water of 5.0 mg L^−1^ [4]. Zn (II) plays a regulatory role in the immune system of the human body, maintaining normal physiological functions in adults, promoting the normal development of children, and promoting the healing of ulcers. It is often used to treat children with anorexia, malnutrition and slow growth, and can also be used to treat hair loss, rashes, mouth ulcers and gastritis. An excess intake of Zn (II) can cause astringency, thirst, a tight chest, dry cough, headaches, dizziness, high fever and chills, among other symptoms. Zn (II) dust is irritating to the eyes and when orally ingested affects the gastrointestinal tract. With this in mind, there are many treatment technologies available to purify water and wastewater contaminated with such heavy metals. The most common methods for removing metal ions from wastewater include chemical precipitation [5,6], extraction [7], redox [8], dialysis [9], electrodialysis [10], electrolytic extraction [11], reverse osmosis [12,13], ion exchange [14,15], adsorption [16,17,18,19,20], ion flotation [21], stripping [22], coagulation flocculation [23], precipitation [24,25] and chelation [26]. Among them, adsorption is widely used as an environmentally friendly method, especially for metal ions that cannot be removed using other techniques. Selective adsorption can also be carried out using biomass materials, such as inorganic clay, activated carbon or polymer resins.

Lignocellulose (LCE) is widely used as a natural biodegradable raw material in the preparation of various biomass absorbent [27]. As the most abundant renewable resource in nature, LCE is widely used in the research and development of new materials, not only because it is cheap and easy to obtain, but more importantly, because it contains lignin, cellulose and hemicellulose. These three components have different properties and can thus be used to change the properties of other materials, whether isolated or used directly [28]. The cellulose, lignin and hemicellulose in LCE are tightly entangled, which results in LCE having a high molecular weight and being difficult to combine with other materials [29,30]. To reduce the molecular weight of LCE and free up functional groups to facilitate the reaction of LCE with other materials, chemical [31] and mechanical methods [32,33,34], as well as a combination of the two, have been employed.

Magnesium aluminum silicate (MOT), the most common layered silicate, is commonly used to prepare absorbents due to its high cation exchange capacity (CEC), surface area, surface reactivity, adsorption performance and low cost [35,36]. MOT has a nanoscale flat sheet layer structure, with this special structure leading to the sheets of MOT being permanently negatively charged, meaning that it exhibits strong exchangeability and adsorption toward positively charged metal cations. The sheet bonding strength of MOT is very weak; thus, it is highly likely that its sheet layers are translated or even peel off, meaning that MOT also exhibits intercalation properties. However, the adsorption capacity of MOT is insufficient, meaning that it cannot be used in large-scale applications. To form MOT absorbent, a monomer or polymer is inserted into the sheet structure of MOT to break its sheet structure. Therefore, to increase its adsorption capacity for Zn (II) cations, MOT can be reacted with nanosized LCE (nano-LCE) to form a polymer gel adsorbent via the attachment of the absorptive functional groups of nano-LCE to the structure of the MOT. Lignocellulose has a large number of hydroxyl groups on its surface, and its fibres are tightly bound via hydrogen bonding, meaning that it exhibits strong polarity and water absorption. The hydrophilic polarity of the lignocellulose surface means that it adsorbs moisture and dissolved substances from water. When a polymer gel is formed from nano-LCE and MOT, nano-LCE is dispersed in the MOT layers, wherein a large number of intermolecular amino bonds are formed between the hydroxyl groups of MOT and the hydroxyl groups of the nano-LCE between the sheets, enhancing the forces between the fibres to form a structurally stable polymer gel material. In this polymer gel, the adsorption performance and the electronegativity of MOT increase the overall adsorption capacity toward Zn (II) cations. Compare with the same type of clay mineral polymer composite material [37,38,39]. This paper uses dissolution and ultrasonic cavitation effects to disperse the LCE, and then insert the dispersed nano-LCE between the magnesium-aluminum silicate clay sheets to re-polymerize the layers to form a stable Three-dimensional network structure similar to that of wood cell walls. Structure to obtain a super absorbent polymer gel with high specific surface area and multiple adsorption sites.

## 2. Materials and Methods

### 2.1. Materials

Zinc oxide (ZnO, analytical grade reagent, AR) was purchased from Sinopharm Chemical Reagent Co., Ltd. (Beijing, China). Sulfuric acid (H_2_SO_4_, AR) was purchased from Sinopharm Chemical Reagent Co., Ltd. (Beijing, China). Sodium hydroxide (NaOH, AR) was purchased from Tianjin Fengchuan Chemical Reagent Co., Ltd. (Tianjin, China). MOT (CEC of 100 meq·100 g^−1^) was produced by Zhejiang Fenghong Clay Chemical Co., Ltd. (Huzhou, China). LCE (SAM-100) was produced by Beijing Huaduo Biotech, Ltd. (Beijing, China). All solutions used in the experiments were prepared using distilled water.

### 2.2. Preparation of the LCE-MOT Polymer Gel

LCE (0.5 g) was weighed into a 50 mL conical flask containing a 25% solution of NaOH (15 mL), which was then placed in a temperature-controlled magnetic stirrer (HJ-6, Changzhou Guohua Instrument Co., Ltd., Changzhou, China) The mixture was then stirred at 50 °C for 30 min to fully expose the LCE to NaOH. Then, MOT (0.5 g) was placed in a 50 mL beaker, to which 15 mL of deionised water was added, and the mixture was stirred at room temperature (23–27 °C) for 30 min. When the MOT was sufficiently dispersed in the deionised water it was assumed that MOT and LCE were mixed, and the resultant mixture was stirred on a temperature-controlled magnetic stirrer at 50 °C for 4 h. After this time, the product was washed with deionised water until pH 7 was reached, then dried at 85 °C for 4 h (DZF-6210, Shanghai Yiheng Scientific Instrument Co., Ltd., Shanghai, China), before being pulverised into a powder, and sieved through a 200-size mesh.

### 2.3. Preparation of Nano-LCE

A 20 wt.% solution of NaOH (250 mL) was prepared and poured into a 250 mL beaker. Then, to this, LCE (0.5 g) was gradually added, and the mixture was slowly stirred with a glass rod until a suspension was formed. The stirred suspension was placed in a SM-1200D ultrasonic cell pulveriser (Nanjing Shunma Co., Ltd., Nanjing, China) and treated at 1080 W for 150 min, before being removed and centrifuged to obtain the target nano-LCE material.

### 2.4. Preparation of the Nano-LCE-MOT Polymer Gel

The prepared nano-LCE was centrifuged and re-dispersed in a 25% solution of NaOH (15 mL) and added to a 50 mL Erlenmeyer flask. Then, the same experimental conditions used in the preparation of the LCE-MOT polymer gel were used to prepare the nano-LCE-MOT polymer gel. After the preparation was completed, the product was freeze-dried and used for subsequent adsorption experiments.

### 2.5. Adsorption Experiments

A Zn (II) ion solution (100 mL) was prepared for adsorption and added to a 250 mL milled Erlenmeyer flask. Hydrochloric acid (HCl) or NaOH (0.1 mol/L) was then used to adjust the pH of the solution to the experimental target value. The adsorbent (0.05 g of the LCE-MOT polymer gel or nano-LCE-MOT polymer gel) was added to the ionic solution, and the flask was then sealed with a glass stopper and placed in a thermostatic shaker (TED, Tianjin Taisite Instrument Co., Ltd., Tianjin China). The adsorption temperature and time were set to complete the adsorption experiments at a constant speed of 120 rpm. After the adsorption process was completed, the resultant mixture was centrifuged at 3000 rpm for 7 min. The Zn (II) concentration of the supernatant was measured using the xylenol orange complex colorimetric method, and the colourised Zn (II) ion solution was then passed through a dual-beam ultraviolet–visible spectrophotometer (TU-1901, Beijing General Analysis General Instruments Co., Ltd., Beijing, China) to measure the absorbance of the Zn (II) complex. A linear regression equation (y = 0.3297 x + 0.409, R^2^ = 0.9998) was then used to determine the concentration of the Zn (II) solution and the amount of Zn (II) adsorbed on the adsorbent. Taking experimental errors and averages into account, three independent replicate experiments confirmed that the Zn (II) removal was reproducible under the same conditions. The amount of metal adsorbed (qt) at equilibrium at time t was calculated using Equation (1):

(1) $$q_{t}=\frac{\left(C_{0}-C_{t}\right)\times V_{0}}{m_{0}}$$

where q_t_ (mg/g) is the capacity of the adsorption at time t; C_0_ and Ct (mg/L) are the initial concentration and concentration of Zn (II) at time t, respectively; V_0_ (L) is the volume of the Zn (II) ion solution and m_0_ (g) is the mass of the adsorbent.

### 2.6. Desorption Experiments

HCl, HNO_3_, H_2_SO_4_ and NaOH solutions used as desorption reagents were prepared at different concentrations. Saturated adsorbent (0.05 g) was weighed into a 250 mL conical flask, to which 50 mL of the desorption reagents were added, and ultrasonic desorption was carried out for a certain time at a certain temperature until desorption equilibrium was reached. The suspension was then centrifuged, and the metal ion concentration in the supernatant of each desorption experiment was tested using the previously mentioned method. The desorption experiments were conducted three times in the same manner, and the desorption capacity at equilibrium (q_t,2_) was calculated using the following equation:

(2) $$q_{t,2}=\frac{C_{t,2}\times V_{2}}{m_{2}}$$

where q_t,2_ (mg/g) is the amount of Zn (II) desorbed at desorption time t (min); C_t,2_ (mg/L) is the concentration of each metal ion in solution at time t (min); V_2_ (mL) is the volume of the desorption agents and m_2_ (g) is the final mass of the adsorbents after release of the metal ions.

Adsorption/desorption cycling tests were carried out to investigate the reusability of the nano-LCE-MOT and LCE-MOT. The nano-LCE-MOT and LCE-MOT were washed until they were restored to their original states after the first batch of adsorption experiments and were then used in the subsequent adsorption experiments. The regenerated nano-LCE-MOT and LCE-MOT were then used in three consecutive cycles under identical conditions and the residual Zn (II) in solution after each adsorption/desorption cycle was measured using the previously mentioned method, and the adsorption/desorption capacity was calculated using the above equations.

## 3. Results

### 3.1. Characterisation of the Materials

The physicochemical properties of the nano-LCE-MOT polymer gel were investigated using different characterisation techniques. The specific surface area, pore structure and pore size of nano-LCE-MOT were measured from nitrogen (N_2_) adsorption–desorption isotherms at −77 °C using an ASAP 2020 surface area analyser (Micromeritics, South Bend, IN, USA). The specific surface area was measured using the Langmuir and Brunauer–Emmet–Teller (BET) methods. Chemical characterisation of the functional groups of TL and TLM was carried out using a spectrophotometer (Thermo Nicolet corporation, Madison, WI, USA) over a scan range of 4000–400 cm^−1^. The powder X-ray diffraction (PXRD) pattern of the TLM was recorded using a diffractometer (X’pero PRO, Armelo, The Netherlands) equipped with a Kα radiation source in the 2θ angle range of 2–15°, operated at 40 kV and 30 mA. Scanning electron microscopy (SEM) of TLM was performed using HITACHI S-4800 (Tokyo, Japan) and S-3400/N Oxford-XMasN (HORIBA, Kyoto, Japan) microscopes. Meanwhile, transmission electron microscopy (TEM, JEM-2010, JEOL, Tokyo, Japan) was carried out at 75–100 kV to investigate the morphology and structure of TLM.

#### 3.1.1. N_2_ Adsorption–Desorption Isotherms of the Nano-LCE-MOT Polymer Gel

**Figure 1:**
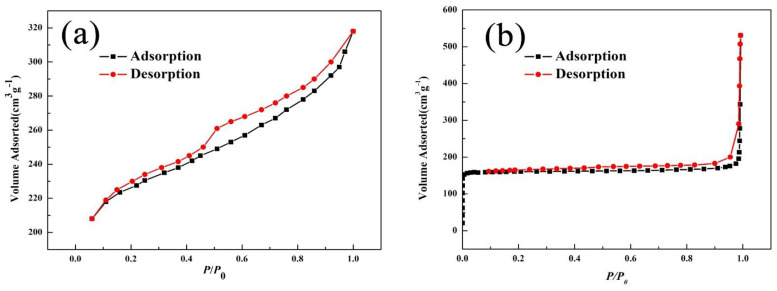
(**a**), Adsorption-desorption curves of N_2_ on lignocellulose magnesium aluminum silicate polymer gel (LCE-MOT) LCE-MOT. (**b**), Adsorption-desorption curves of N_2_ on Nano-LCE-MOT.

The N_2_ adsorption/desorption isotherms of LCE-MOT and nano-LCE-MOT are shown in Figure 1a,b, respectively. From Figure 1a,b, it can be observed that LCE-MOT exhibits a type II-shaped isotherm, with a H_3_ hysteresis loop in the middle of the curve, while nano-LCE-MOT exhibits a type IV-shaped isotherm, with a H_4_ hysteresis loop in the middle of the curve, indicating that there are a lot of slit pores in the nano-LCE-MOT, which is different from particle accumulation and is a result of its layered structure [40]. According to Table 1, the average pore size of LCE-MOT is around 31.82 nm, whereas that of nano-LCE-MOT is smaller at around 6.17 nm. Meanwhile, the specific surface area of LCE-MOT was calculated using the Langmuir method (378.06 m^2^/g) and the BET (213.50 m^2^/g) methods, and that of nano-LCE-MOT was found to be higher at 701.80 m^2^/g (Langmuir method) and 532.74 m^2^/g (BET method). The experimental results indicate that the specific surface area, pore volume and mean pore size of nano-LCE-MOT were enhanced to varying degrees after its nanosizing treatment, with its increased parameters enhancing its adsorption capacity toward Zn (II) [37].

#### 3.1.2. FTIR Analysis of the Materials

**Figure 2:**
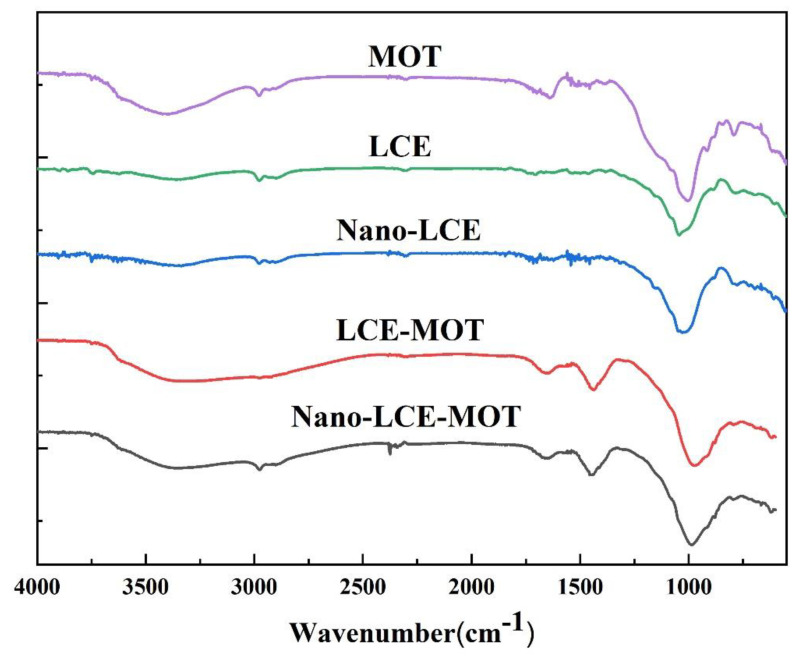
FTIR spectra of MOT, LCE, Nano-LCE, LCE-MOT, Nano-LCE-MOT.

The characteristic chemical structures of MOT, LCE, nano-LCE, LCE-MOT and nano-LCE-MOT were characterised by FTIR spectroscopy in the wavenumber range of 4000–400 cm^−1^. Figure 2 shows the FTIR spectrum of LCE, which features peaks that can be attributed to -OH bending (3366 cm^−1^), the –OH stretching vibration of -COOH (1462 cm^−1^) and the -C-O-C- stretching vibration of cellulose and hemicellulose (1050 cm^−1^) [41], while after nanosizing treatment, the absorption peaks were shifted to 3360, 1474 and 1037 cm^−1^, respectively, and a new peak attributed to -C=O appeared at 1622 cm^−1^ [42], indicating that the active groups of lignocellulose were exposed after the nanosizing treatment. From the results shown in Figure 2, it is inferred that the nanosizing treatment of LCE does not change its chemical structure, it only reduces its size to nanosized, thus enhancing its adsorption capacity toward Zn (II) from effluent.

Upon the intercalation of LCE and nano-LCE into the interlayers of MOT, the peak of -OH (3406 cm^−1^) shifted to a higher wavenumber of 3468 cm^−1^ for LCE-MOT and 3438 cm^−1^ for nano-LCE-MOT, and the carbonyl -OH absorption peak shifted to 1464 and 1456 cm^−1^, respectively. The telescopic vibration adsorption peaks at 909 and 786 cm^−1^ can be attributed to -Al-O-H and -Si-O in in the structure of MOT, respectively, and they were clearly abated, as is evident from the spectra of LCE-MOT and nano-LCE-MOT [43]. As shown in the FTIR spectra, LCE and nano-LCE intercalate into the interlayers of MOT, and there are many different types of adsorption sites on the surface of LCE-MOT and nano-LCE-MOT, such as -Al-O, -Si-O, -C-O-C-, -C=O and -OH, which enhance their capacity to adsorb heavy metals [44].

#### 3.1.3. PXRD Analysis of the Materials

**Figure 3:**
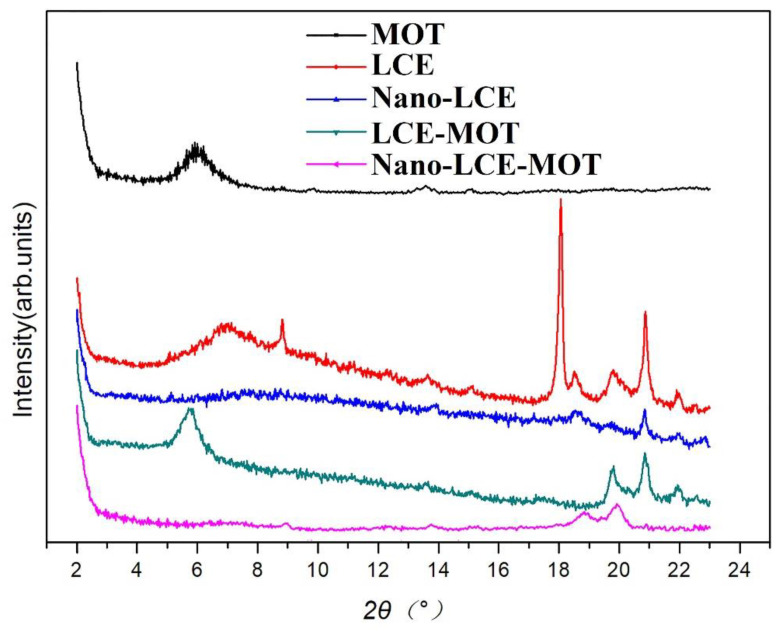
XRD patterns of MOT, LCE, Nano-LCE, LCE-MOT, Nano-LCE-MOT.

As shown in Figure 3, the PXRD pattern of MOT exhibits a characteristic diffraction peak at 5.92°, which indicates that it maintains a relatively complete crystal structure and has a typical nanomaterial structure [45]. After the intercalation reaction of MOT with LCE and nano-LCE, the characteristic diffraction peak of MOT disappeared, indicating that the layered structure of MOT is destroyed during the intercalation process as LCE and nano-LCE enter into the layers of MOT, forming an intercalation stripped nanostructure. Apparently, in the intercalation process, nano-LCE inflicts greater damage on the layered structure of the MOT, so the specific surface area of nano-LCE-MOT is higher than LCE-MOT.

#### 3.1.4. SEM Analysis of Nano-LCE-MOT and LCE-MOT

**Figure 4:**
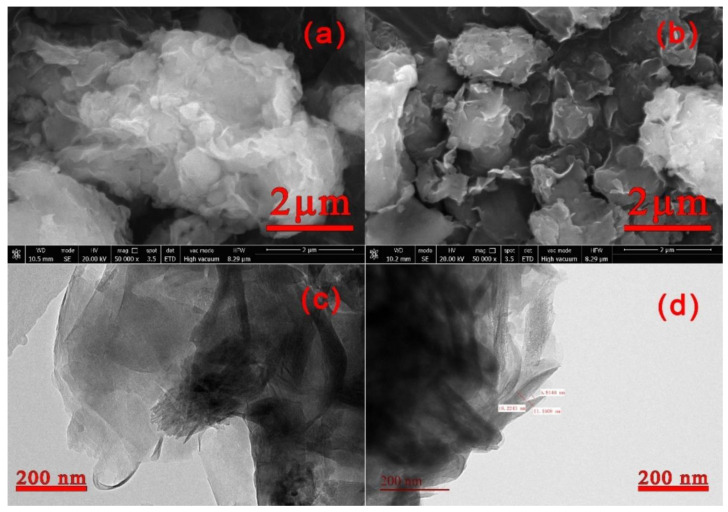
(**a**), SEM of LCE-MOT. (**b**), SEM of Nano-LCE-MOT. (**c**), TEM of LCE-MOT. (**d**), TEM of Nano-LCE-MOT.

To further investigate the microstructures of LCE-MOT and nano-LCE-MOT, their intercalation and exfoliation were analysed, and their surface morphologies were observed by SEM. It can be seen from Figure 2 and Figure 4a that the surfaces of LCE-MOT and nano-LCE-MOT are very rough and dispersed, and their lamellar structures exhibit curly shapes. The results indicate that nano-LCE destroys the lamellar crystal structure of MOT and enters its interlayer domain, which is consistent with the PXRD results.

#### 3.1.5. TEM Analysis of Nano-LCE-MOT and LCE-MOT

The morphologies and structures of LCE-MOT and nano-LCE-MOT were investigated using TEM. From the results, it can be seen that LCE and nano-LCE intercalate into the MOT layers and destroy the MOT structure. In Figure 4c,d, the shaded part is the lamellar structure of MOT, and the black rods are LCE (c) and nano-LCE (d). As the particle size of nano-LCE is smaller than that of LCE, it more easily enters into the MOT lamellae, which means that the black area in Figure 4d is more obvious than that in Figure 4c, so the specific surface area of nano-LCE-MOT is the highest of the polymer gel materials, and there are more active adsorption sites for the adsorption of heavy metals. Thus, the MOT lamellar crystals are stretched or stripped to form intercalation stripped materials.

### 3.2. Adsorption Studies

#### 3.2.1. Effect of the Initial Zn (II) Concentration

**Figure 5:**
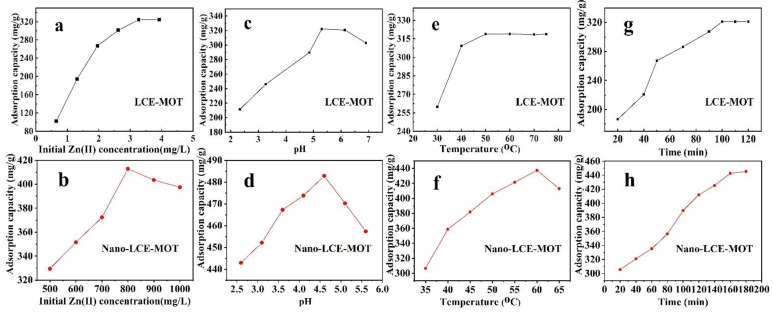
Effect of Zn (II) initial concentration on adsorption capacity of LCE-MOT (**a**) and Nano-LCE-MOT (**b**). Effect of pH on adsorption capacity of LCE-MOT (**c**) and Nano-LCE-MOT (**d**). Effect of Temperature on adsorption capacity of LCE-MOT (**e**) and Nano-LCE-MOT (**f**). Effect of Time on adsorption capacity of LCE-MOT (**g**) and Nano-LCE-MOT (**h**).

Figure 5a,b show the effect of the concentration of Zn (II) on the adsorption equilibrium behaviour of LCE-MOT and nano-LCE-MOT as Zn (II) adsorption materials. It is shown that the adsorption capacity of Zn (II) using LCE-MOT (0.65–4.58 g/L for Zn (II)) and nano-LCE-MOT (500–1000 mg/L for Zn (II)) increased in line with a higher initial metal concentration until maximum capacity was reached, but the maximum adsorption capacity of nano-LCE-MOT was higher than that of LCE-MOT, with a maximum adsorption capacity of 412.83 mg/g. The adsorption capacities of LCE-MOT and nano-LCE-MOT exhibited maximum adsorption at initial Zn (II) concentrations of 3.27 and 800 mg/L, respectively. When the concentration was lower at the beginning of the experiment, the adsorption of heavy metals was on the exterior surface of LCE-MOT and nano-LCE-MOT, yet with increasing concentration, the heavy metal ions were able to overcome the resistance due to mass transfer from the aqueous to solid phase and subsequently penetrated into the interior of the structure, giving rise to increasing numbers of adsorption sites on LCE-MOT and nano-LCE-MOT. The specific surface area of nano-LCE-MOT is therefore higher than that of LCE-MOT, so it has a higher number of adsorption sites than LCE-MOT, which leads to extra adsorption sites for Zn (II) to occupy on the surface of nano-LCE-MOT [46]. It is clear that the adsorption capacity did not change significantly after an additional increase in the metal concentration due to there being no more sites available for heavy metal adsorption on the LCE-MOT and nano-LCE-MOT surfaces.

#### 3.2.2. Effect of pH

Figure 5c,d show the effects of the starting pH of the solution on the adsorption of Zn (II). As noted from the figures, the pH has a marked impact on the adsorption process of LCE-MOT and nano-LCE-MOT toward Zn (II). The adsorption capacities of Zn (II) were found to be enhanced with increasing pH, and then slightly decreased upon a further increase in the pH. The capacity for the adsorption of Zn (II) rose from 215 to 351 mg/g for LCE-MOT and 443 to 481 mg/g for nano-LCE-MOT upon an increase in pH. Heavy metal adsorption is dependent on the interactions among the heavy metal ions and the active sites on the exterior of LCE-MOT and nano-LCE-MOT, which are impacted by the solution pH. At low pH, the competition between the H^+^ ions and the adsorption sites on the LCE-MOT and nano-LCE-MOT surfaces became more intense in line with an increase in the H^+^ concentration, thus leading to a decrease in adsorption capacity [47]. At low pH, Zn (II) exists in the form of a dissolved metal, whereas at pH values of above approximately, Zn may precipitate out as ZnOH and Zn (OH)_2_, decreasing the percentage of adsorption [48]. Comparing the adsorption behaviour of LCE-MOT and nano-LCE-MOT toward Zn (II), the adsorption capacity of nano-LCE-MOT toward Zn is much higher than that of LCE-MOT, even at lower pH, where similar behaviour is observed. The reason for this might be because as LCE undergoes nanosizing treatment, there are more adsorption sites on the surface of nano-LCE-MOT, meaning that under low adsorption conditions at low pH, although the H^+^ ions compete with the active sites, nano-LCE-MOT still has other sites at which Zn (II) can adsorb. Meanwhile, the specific surface area of LCE-MOT is lower than that of nano-LCE-MOT, resulting in the adsorption capacity of nano-LCE-MOT being higher than that of LCE-MOT. At higher pH values, the adsorption of Zn (II) ions decreased to different degrees. Therefore, pH 5.30 for LCE-MOT and 4.52 for nano-LCE-MOT were selected as the pH values for use in subsequent experiments.

#### 3.2.3. Effect of Adsorption Temperature

Figure 5e,f show the adsorption characteristics of LCE-MOT and nano-LCE-MOT at different temperatures, from which it can be seen that with an increase in the temperature the adsorption capacities of LCE-MOT and nano-LCE-MOT increased gradually and reached peak adsorption capacities of 318 and 430 mg/g, respectively, demonstrating that an increase in temperature is more favourable for improving the adsorption of the materials toward Zn (II). With an increase in the adsorption temperature, the rate at which the heavy metal ions pass through the material pores and the diffusion rate on the materials surface increase, which leads to an increase in the adsorption capacity [49]. At the same time, with an increase in temperature, nano-LCE-MOT exhibits higher energy, and the numbers of collisions between the active sites and heavy metal ions on its surface increase, which accounts for the adsorption capacity of nano-LCE-MOT toward Zn (II) being higher than that of LCE-MOT.

#### 3.2.4. Effect of Adsorption Time

Figure 5g, h showsthe effect that the adsorption time has on the removal of Zn (II), from which it can be seen that Zn (II) adsorption follows a steady increasing trend with time and is rapid on the two materials in the early process of adsorption. As presented in Figure 5g,h, the adsorption capacities of the Zn (II) ions increased rapidly from 180 to 330 mg/g on LCE-MOT and 300 to 440 mg/g on nano-LCE-MOT, respectively, in line with an increase in the adsorption time. In the initial stage of the adsorption process, there is a big difference in the concentration between the internal and external heavy metal ions of the adsorption material, so the driving force of the diffusion of heavy metal ions into the material is large at the initial stage of adsorption. With an increase in the adsorption time, the number of available adsorption sites on the material gradually decreases until saturation is reached, and the adsorption capacity then reaches equilibrium [50]. After the nanosizing treatment of nano-LCE-MOT, the material has more adsorption sites on its surface, which means that nano-LCE-MOT takes longer than LCE-MOT to reach adsorption equilibrium. Therefore, the adsorption capacity of nano-LCE-MOT is higher than LCE-MOT toward Zn (II), and the time taken to reach adsorption equilibrium is slightly longer.

#### 3.2.5. Adsorption Kinetics and Mechanism

Prediction of the adsorption rates provides important information regarding adsorption mechanisms. To this aim, to evaluate the adsorption mechanisms of nano-LCE-MOT and LCE-MOT, the experimental data at various adsorption times corresponding to the changes in adsorption capacity were fitted using pseudo-first order (Equation (3)) and pseudo-second order (Equation (4)) models:

(3) $$\ln\left(q_{\mathrm{et}}-q_{t}\right)=\ln q_{\mathrm{et}}-k_{1}t$$

(4) $$\frac{t}{q_{t}}=\frac{t}{q_{e}}+\frac{1}{k_{2}q_{e}^{2}}$$

where q_e_ and q_t_ are the amounts of heavy metal ions adsorbed (mg/g) at equilibrium and at time t (min), respectively; k_1_ (min^−1^) is the pseudo-first-order rate constant and k_2_ [g·(mg/min) ^−1^] is the rate constant of the pseudo-second-order adsorption kinetic equation.

**Figure 6:**
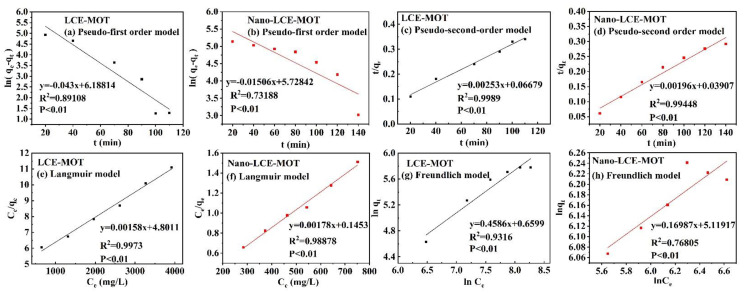
(**a**–**d**) Adsorption kinetics equation fitting curves of the experimental data. (**e**) The Langmuir; (**g**) Freundlich; models for the adsorption of Zn (II) ions by LCE-MOT. (**f**) The Langmuir; (**h**) Freundlich; models for the adsorption of Zn (II) ions by Nano-LCE-MOT.

The validity of the pseudo-first order and pseudo-second-order models was tested by fitting the data to a straight line, with the results shown in Figure 6a–d. The respective parameters for the reaction kinetic and determination coefficients are given in Table 2. Through the fitting of the data to the equations (Table 2), the adsorption of Zn (II) by nano-LCE-MOT and LCE-MOT was found to fit the pseudo-second-order model. From the data, it could be inferred that there was almost no impact on the adsorption rate by mass diffusion at a constant adsorption time according to the pseudo-second-order kinetic reaction mechanism. This demonstrates that the adsorption mechanisms of nano-LCE-MOT and LCE-MOT are controlled primarily by chemisorption [51]. Apparently, the maximum adsorption capacity of nano-LCE-MOT toward Zn (II) was higher than that of LCE-MOT, which might be related to the nanosizing treatment of LCE, which increases the number of adsorption sites on the surface of nano-LCE-MOT that can combine with Zn (II).

#### 3.2.6. Adsorption Modelling

To better understand the ion adsorption processes that occur on nano-LCE-MOT and LCE-MOT, the Langmuir (Equation (5)) and Freundlich (Equation (6)) isotherms were applied to fit the adsorption equilibrium data obtained from the batch adsorption experiments. Figure 6e–h shows the fitting of the data to the isotherms, and the correlation coefficient values, among other parameters, are presented in Table 3. From the results, the Langmuir model was found to be more suitable than the Freundlich model. The Langmuir model makes the assumption that the adsorption in the monolayer of the adsorbent takes place when the adsorbent has a uniform exterior structure, in which the binding sites have identical affinity toward adsorption and there is zero interaction among the adsorbent binding sites. According to the intrinsic features of the Langmuir model, it was revealed that the adsorption of Zn (II) by nano-LCE-MOT and LCE-MOT was confined to the monolayers created between the adsorbents and heavy metals, with zero additional same-plane interactions on the surface of the adsorbent by the adsorbate, in a multilayer coverage fashion [52]. The maximum theoretical adsorption capacities of nano-LCE-MOT and LCE-MOT at their optimal temperatures were found to be 326.08 and 561.80 mg/g, respectively, as shown in Table 3. Meanwhile, as also shown in Table 3, compared with the previously reported adsorbents, nano-LCE-MOT and LCE-MOT exhibit higher adsorption capacity toward Zn (II), which illustrates that nano-LCE-MOT and LCE-MOT are excellent adsorbents for the adsorption of Zn (II) from aqueous media. However, maximum adsorption capacity of nano-LCE-MOT is higher than that of LCE-MOT, which proves that the nanosizing treatment tends to improve the intercalation reaction effect and enhance the adsorption capacity of the material. It was further proved that chemisorption affects the adsorption process:

(5) $$\frac{C_{e}}{q_{e}}=\frac{1}{K_{L}q_{\max}}+\frac{C_{e}}{q_{\max}}$$

(6) $$\ln q_{e}=\ln K_{f}+\frac{1}{n}\ln C_{e}$$

where K_L_(L/mg) is the Langmuir constant related to the adsorption capacity; q_max_ (mg/g) is the monolayer saturation adsorption capacity; 1/n is the value used to indicate the heterogeneity of the interface; K_f_ is the Freundlich constant, and Ce (mg/L) is the concentration of metal ions at equilibrium.

### 3.3. Desorption and Regeneration Studies

#### 3.3.1. Effects of Various Desorption Reagents

**Figure 7:**
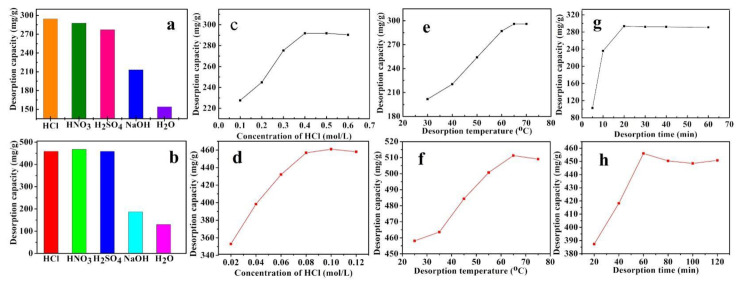
Effects of various desorption reagents on desorption capacity of LCE-MOT (**a**) and Nano-LCE-MOT (**b**). Effects of desorption agents’ concentration on desorption capacity of LCE-MOT (**c**) and Nano-LCE-MOT (**d**). Effects of desorption temperature on desorption capacity of LCE-MOT (**e**) and Nano-LCE-MOT (**f**). Effects of desorption time on desorption capacity of LCE-MOT (**g**) and Nano-LCE-MOT (**h**).

The desorption capacities of nano-LCE-MOT and LCE-MOT were investigated using HCl, HNO_3_ and H_2_SO_4_ as proton exchange desorption reagents and NaOH as a chelating desorption reagent to regenerate the materials. Figure 7a,b show the effects of the different desorption reagents on the desorption capacity of nano-LCE-MOT and LCE-MOT. It can be seen from the results of the desorption experiments that both the desorption processes of Zn (II) from Zn (II)-loaded nano-LCE-MOT and Zn (II)-loaded LCE-MOT were higher using HCl than the other desorption reagents. Hence, the desorption of Zn (II) from nano-LCE-MOT and LCE-MOT was performed using HCl in the subsequent regeneration experiments.

#### 3.3.2. Effects of Desorption Agent Concentration

The desorption capacity was found to be related to the concentration of the desorption regents. As show in Figure 7c,d, the effects that different concentrations of HCl have on the desorption of Zn (II)-loaded nano-LCE-MOT and Zn (II)-loaded LCE-MOT were investigated. It can be seen from the data that the desorption capacity increases in line with an increase in the concentration of HCl until the system reaches desorption equilibrium. Also, the desorption capacity of nano-LCE-MOT was found to be higher than that of LCE-MOT and that maximum desorption of Zn (II)-loaded nano-LCE-MOT could therefore be reached using a lower concentration of HCl. The results of the experiments infer that as the proton (H^+^) concentration is lower at a lower concentration of HCl, the H^+^ are completely replaced, resulting in nano-LCE-MOT and LCE-MOT losing their absorbability. While, in the presence of a high concentration of H^+^ the adsorbents are protonated, which means that the adsorption site for Zn (II) are occupied by H^+^, meaning that there are no increases in the desorption capacities of Zn (II)-loaded nano-LCE-MOT and Zn (II)-loaded LCE-MOT. The maximum desorption values of Zn (II)-loaded nano-LCE-MOT and Zn (II)-loaded LCE-MOT reached 456.81 and 291.88 mg/g for Zn (II) at HCl concentrations of 0.08 and 0.4 mol/L, respectively.

#### 3.3.3. Effects of Desorption Temperature

As shown in Figure 7e,f, the desorption capacities of the absorbents increased in line with the temperature, which might be a result of more H+ ion activity at higher temperature, meaning that the H+ compete with Zn (II) on the surface of Zn (II)-loaded nano-LCE-MOT and Zn (II)-loaded LCE-MOT, thus leading to an increase in the desorption rate. While, upon a further increase in the temperature, the active sites might be destroyed if the temperature is too high [53], hence leading to a slight decrease in the desorption capacities of Zn (II)-loaded nano-LCE-MOT and Zn (II)-loaded LCE-MOT.

#### 3.3.4. Effects of Desorption Time

As shown in Figure 7g,h, the best desorption times of nano-LCE-MOT and LCE-MOT were 60 and 20 min, respectively. With an increase in the desorption time, the desorption capacity first increased and then decreased. In the initial stage of desorption, H+ gradually replaces the Zn (II) adsorbed on the material, therefore meaning that there is a gradual increase in the adsorption capacity. After a certain adsorption time, the material continues to adsorb Zn (II), so as to reach adsorption–desorption equilibrium.

#### 3.3.5. Regeneration and Reuse

The recovery of heavy metals adsorbed on the nano-LCE-MOT and LCE-MOT surfaces is of great significance toward improving the economy of nano-LCE-MOT and LCE-MOT. Table 4 shows the data on the reusability of nano-LCE-MOT and LCE-MOT investigated over three adsorption/desorption cycles. The adsorption capacities of nano-LCE-MOT and LCE-MOT were observed to decrease gradually with the number of cycles, which might be because the adsorption sites on the surfaces of nano-LCE-MOT and LCE-MOT were destroyed at low pH during the regeneration cycles. The results show that nano-LCE-MOT could be recycled up to three times for the adsorption of Zn (II) with very little loss in efficiency, while LCE-MOT could be recycled twice, proving that the nanosizing treatment enhances the reusability of the adsorbent.

#### 3.3.6. Adsorption Capacity of Lignocellulose-Based Materials to Zn (II)

Table 5 lists four lignocellulose-based adsorbents for comparison with this article. It can be seen from the table that nano-LCE-MOT has a good adsorption effect on Zn (II). It can be concluded that the material can be used to adsorb Zn (II).

## 4. Discussion

**Figure 8:**
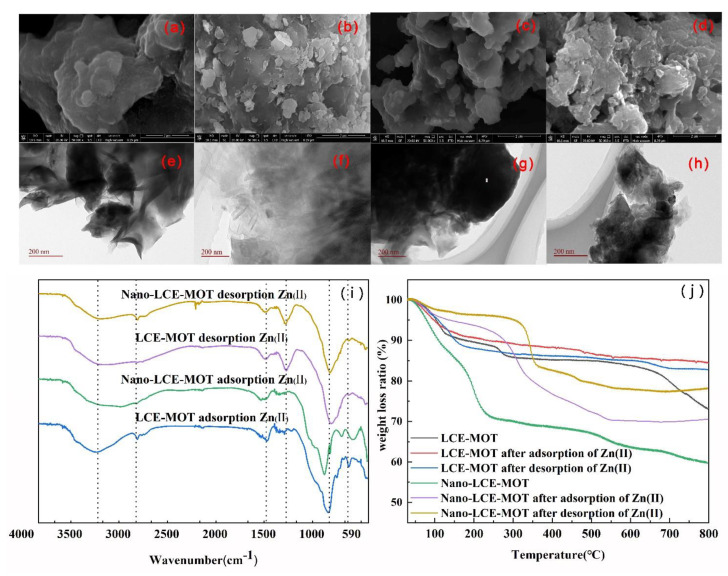
SEM of LCE-MOT after adsorption (**a**) and desorption (**b**) of Zn (II). SEM of Nano-LCE-MOT after adsorption (**c**) and desorption (**d**) of Zn (II). TEM of LCE-MOT after adsorption (**e**) and desorption (**f**) of Zn (II). TEM of Nano-LCE-MOT after adsorption (**g**) and desorption (**h**) of Zn (II). (**i**) FTIR of Nano-LCE-MOT and LCE-MOT adsorption and desorption Zn (II). (**j**) TG of Nano-LCE-MOT and LCE-MOT adsorption and desorption Zn (II).

The adsorption mechanism of Zn (II) on nano-LCE-MOT and LCE-MOT was clarified by FTIR spectroscopy, TGA, SEM, TEM and EDX-mapping analysis. As shown in the FTIR spectra in Figure 8i, nano-LCE-MOT and LCE-MOT exhibit peaks at 3438 and 3468 cm^−1^, respectively, which can be attributed to the -O-H stretching of the hydroxyl groups. As shown in the data, after adsorption, these peaks shifted to lower wavenumber values of 3374 and 3349 cm^−1^ respectively, indicating that the -O-H groups of nano-LCE-MOT and LCE-MOT participated in the adsorption process, which in turn demonstrates the involvement of oxygen in the chemisorption of Zn (II) in the subsequent complexation process. The characteristic absorption peaks at approximately 1690 cm^−1^ for LCE-MOT and 1646 cm^−1^ for nano-LCE-MOT can be attributed to -C=O stretching in the carboxylic organic acid groups of the materials, which were shifted to lower wavenumber values of 1687 and 1657 cm^−1^ after adsorption and 1657 and 1643 cm^−1^ after desorption, respectively, as the possible result of the oxygen in the -C=O of nano-LCE-MOT and LCE-MOT forming complexes with the heavy metal ions [58]. The -OH in the carboxylic group exhibited characteristic absorption peaks for nano-LCE-MOT and LCE-MOT at 1464 and 1456 cm^−1^, respectively, which disappeared after adsorption, proving that the -C=O bonds in the carboxylic organic acid groups participate in the chemisorption process. These changes in the absorption peaks indicate that the activated sites on the surfaces of nano-LCE-MOT and LCE-MOT contain hydroxyl and carboxylic functional groups, which form new chemical bonds with Zn (II), resulting in some of the peaks shifting and disappearing. Furthermore, although ion exchange and electrostatic attraction may also occur during the adsorption process chemical adsorption is the main adsorption process.

From the TGA results, as the Zn (II) ions are adsorbed on the surfaces of nano-LCE-MOT and LCE-MOT, this has an impact on the weight loss of the adsorbents. The mass loss rate of the nanosizing treated nano-LCE-MOT was higher than that of LCE-MOT, proving that that nanosizing treatment leads to the adsorbent more readily undergoing thermal decomposition. As shown in Figure 8j in the TGA curves of nano-LCE-MOT with adsorbed Zn (II) and LCE-MOT with adsorbed Zn (II), the weight loss ratios of the adsorbents changed when they were loaded with Zn (II) ions. This indicates that Zn (II) strongly adsorbs onto nano-LCE-MOT and LCE-MOT, therefore contributing toward their loss in weight. The experimental results prove that the adsorption processes of Zn (II) on nano-LCE-MOT and LCE-MOT are both physical and chemical in nature, which might be due to the interaction between the oxygen-containing functional groups on the adsorbent surface and Zn (II) taking place via a complexation mechanism [59].

Figure 8a–h show the SEM and TEM images of nano-LCE-MOT and LCE-MOT after the adsorption and desorption of Zn (II). It can be seen from Figure 8a–h that the layered structures of the surfaces of the adsorbents disappear after Zn (II) adsorption, with the appearance of a lot of granular matter on their surface. This indicates that Zn (II) adsorbs at the adsorption sites and pores of the materials, with the materials regaining loose layered structures and pores after Zn (II) desorption [60].

**Figure 9:**
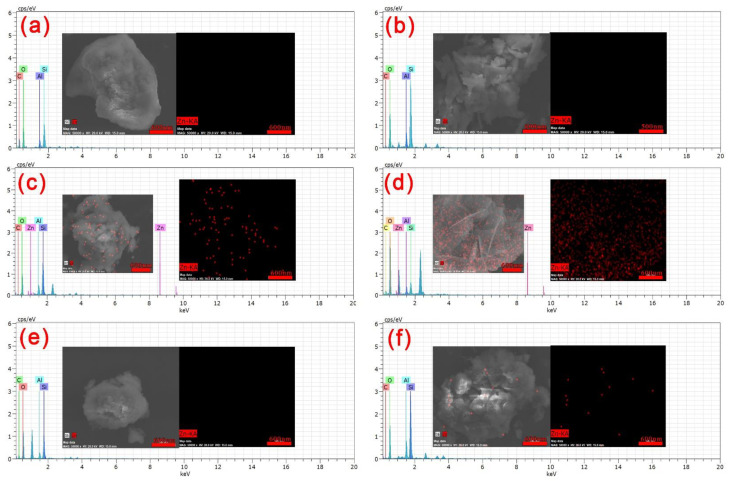
EDX and mapping of LCE-MOT (**a**), LCE-MOT adsorption (**c**) and desorption (**e**) Zn (II).EDX and mapping of Nano-LCE-MOT (**b**), Nano-LCE-MOT adsorption (**d**) and desorption (**f**) Zn (II).

From the EDX data in Figure 9a–f, it can be seen that there are only four elements, C, O, Si and Al, present in nano-LCE-MOT and LCE-MOT. After the adsorption of Zn (II), the peak for Zn (II) is present in the EDX data, and the intensity of the absorption peak of oxygen decreases. From the mapping diagram, it can be seen that the Zn (II) is uniformly distributed on both the LCE-MOT and the nano-LCE-MOT surfaces, with more Zn (II) being obviously distributed on nano-LCE-MOT than on LCE-MOT. Therefore, this means that the adsorption capacity of nano-LCE-MOT toward Zn (II) is higher than that of LCE-MOT. After desorption, the Zn (II) on the surface of the materials is desorbed, and only the signals of the original elements can be once again observed in the EDX data. The oxygen peaks returns to its original intensity, with no signal for Zn (II) observed in the mapping data, which indicates that Zn (II) is completely desorbed, and that oxygen participates in the adsorption process.

## 5. Conclusions

In this study, it was shown that LCE/MOT and nano-LCE/MOT can be used to effectively extract Zn (II) from aqueous solution, with the adsorption capacity toward Zn (II) of nano-LCE/MOT being higher than that of LCE/MOT, reaching 513.48 mg/g. The optimal adsorption of Zn (II) on nano-LCE/MOT occurs at pH 4.52, at 60 °C, with a contact time of 160 min. The optimal adsorption of Zn (II) on LCE/MOT occurs at pH 5.3, at 50 °C, with a contact time of 100 min. Among the evaluated kinetic models and isotherms, the pseudo-second-order adsorption kinetics equation and Langmuir isothermal adsorption model gave the best fit for the Zn (II) adsorption process, suggesting that the adsorption process mainly occurs via monolayer chemical adsorption. Desorption and regeneration experiments revealed that HCl eluted 456.81 mg/g of Zn (II) from Zn-loaded nano-LCE/MOT and 291.88 mg/g of Zn (II) from Zn-loaded LCE/MOT. Overall, these results suggest that nano-LCE/MOT is an excellent adsorbent for the extraction of Zn (II) from aqueous media.

## Figures and Tables

**Table 1:** Surface area and pore structure parameters of MOT, LCE-MOT, Nano-LCE-MOT.

Sample	SBET (m^2^/g)	SLangmuir (m^2^/g)	VMic (cm^3^/g)	RMic (nm)	Rmes (nm)	RAve (nm)
MOT	61.4	137.52	0.024			204.05
LCE-MOT	213.50	378.06	1.051	1.98	45.93	31.82
Nano-LCE-MOT	532.74	701.80	0.25	0.68	173.02	6.17

**Table 2:** R^2^ and constant values for the different adsorption kinetics models of Zn (II) for Nano-LCE-MOT and LCE-MOT.

Materials	Parameters	Pseudo-First Order		Pseudo-Second Order	
LCE-MOT	R^2^	0.8912		0.9989	
	Constants	k_1_	0.0045 min^−1^	k_2_	0.2876 min^−1^
		q_e_	297.01 mg/g	q_e_	327.11 mg/g
Nano-LCE-MOT	R^2^	0.7319		0.9945	
	Constants	k_1_	0.015 min^−1^	k_2_	0.000098 min^−1^
		q_e_	541.10 mg/g	q_e_	510.20 mg/g

**Table 3:** R^2^ and constant values for the different adsorption isotherm models of Zn (II) for Nano-LCE-MOT and LCE-MOT.

Materials	Parameters	Langmuir		Freundlich	
LCE-MOT	R^2^	0.9973		0.9316	
	Constants	K_L_	0.0311 L/mg	K_f_	69.11 L/mg
		q_max_	326.08 mg/g	1/n	0.135
Nano-LCE-MOT	R^2^	0.9888		0.7681	
	Constants	K_L_	0.0012 L/mg	K_f_	167.28 L/mg
		q_max_	561.80 mg/g	1/n	0.1699

**Table 4:** Adsorption/desorption cycles for Zn (II) onto Nano-LCE-MOT and LCE-MOT.

Materials	Adsorption-Desorption Capacity	Adsorption/Desorption Cycles		
		1	2	3
LCE-MOT	Adsorption capacity	324.02 mg/g	317.05 mg/g	280.23 mg/g
	Desorption capacity	296.11 mg/g	267.11 mg/g	145.37 mg/g
Nano-LCE-MOT	Adsorption capacity	520.33 mg/g	503.73 mg/g	497.54 mg/g
	Desorption capacity	507.29 mg/g	497.38 mg/g	490.63 mg/g

**Table 5:** Comparison of adsorption properties of different materials for Zn (II).

Sample	Zn (II) (mg/g)	Author
nano-LCE-MOT	520.33	This work
Thiol-lignocellulose sodium bentonite nanocomposites	357.29	Zhang [37]
Smectite clay	102.04	Sdiri [38]
Silica nanoparticles	49.75	Diab [39]
Sulfhydryl-modified cassava straw	60.24	Deng [54]
Lignocellulose@ activated clay nanocomposite	315.90	Zhang [55]
Functionalized lignocellulose derived from waste biomass	46.49	Dang [56]
Biochars derived from long-root *Eichhornia crassipes*	45.40	Li [57]

## Data Availability

The data presented in this study are available on request from the corresponding author.
